
JOURNAL INFORMATION
==============================
NLM Title Abbreviation: Wirtschaftsdienst
Journal ID: Wirtschaftsdienst
NLM Journal ID: 101086612

Wirtschaftsdienst (Hamburg, Germany : 1949)

ISSN: 0043-6275

ARTICLE INFORMATION
==============================
PMCID: PMC7335924
PMID: 32834154
DOI: 10.1007/s10273-020-2670-y
Article ID: 2670
Article version: 1
Subjects: Zeitgespräch

Europa in der Corona-Krise: Welche Maßnahmen sind nötig und auf welcher Ebene sollten sie ansetzen?

Gros Daniel danielg@ceps.be

grid.22793.3d 0000 0004 0609 4239 Centre for European Policy Studies (CEPS), 1 Place du Congrès, 1000 Brussels, Belgien
Electronic publication date: 2020 Jul 6
Print publication date: 2020
Volume: 100
Issue: 6
First page: 404
Last page: 407
Copyright: © Der/die Autor(en) 2020
License: Open Access Dieser Artikel wird unter der Creative Commons Namensnennung 4.0 International Lizenz veröffentlicht, welche die Nutzung, Vervielfältigung, Bearbeitung, Verbreitung und Wiedergabe in jeglichem Medium und Format erlaubt, sofern Sie den/die ursprünglichen Autor(en) und die Quelle ordnungsgemäß nennen, einen Link zur Creative Commons Lizenz beifügen und angeben, ob Änderungen vorgenommen wurden.
Die in diesem Artikel enthaltenen Bilder und sonstiges Drittmaterial unterliegen ebenfalls der genannten Creative Commons Lizenz, sofern sich aus der Abbildungslegende nichts anderes ergibt. Sofern das betreffende Material nicht unter der genannten Creative Commons Lizenz steht und die betreffende Handlung nicht nach gesetzlichen Vorschriften erlaubt ist, ist für die oben aufgeführten Weiterverwendungen des Materials die Einwilligung des jeweiligen Rechteinhabers einzuholen.
Weitere Details zur Lizenz entnehmen Sie bitte der Lizenzinformation auf http://creativecommons.org/licenses/by/4.0/deed.de.
License URL: https://creativecommons.org/licenses/by/4.0/

issue-copyright-statement: © The Author(s) 2020

==============================

Die Corona-Krise hat die ganze Welt erfasst. Europa wurde besonders hart getroffen. Anfangs hat noch jeder Mitgliedstaat der Europäischen Union nur an sich selbst gedacht. Die Fallzahlen stiegen exponentiell, die Gesundheitssysteme vieler Länder kamen an ihre Grenzen und die Finanzmärkte stürzten ins Chaos. Inzwischen hat sich das Blatt gewendet. Das Virus scheint besiegt, die Finanzmärkte haben sich erholt und auch bei der Wirtschaft gibt es erste Zeichen einer Erholung. Zu dieser Wende hat auch die Einsicht beigetragen, dass diese Krise gemeinsames Handeln verlangt. Diese hat zu dem deutsch-französischen Vorschlag eines europäischen Fonds von 500 Mrd. Euro geführt, der inzwischen von der europäischen Kommission aufgegriffen und erweitert wurde. Die Kernfragen dabei sind: Was für Maßnahmen werden benötigt und auf welcher Ebene sollten die Maßnahmen ansetzen? Ist es sinnvoll, dass nur die Staaten der Eurozone stärker zusammenstehen oder bedarf es der Solidarität innerhalb der gesamten EU27?

Die Corona-Krise ist theoretisch eine symmetrische Krise, denn alle Länder mussten ihre Wirtschaft zeitweilig zum Stillstand bringen. Es steht aber außer Frage, dass die wirtschaftlichen Folgen der Corona-Epidemie verschiedene Mitgliedstaaten mit sehr unterschiedlicher Intensität treffen. In dieser außergewöhnlichen Situation ist Solidarität notwendig. Die Schwächsten, die leider am härtesten getroffen werden, verdienen Hilfe. Die stärkeren Mitgliedstaaten, die helfen können, vor allem also Deutschland, werden selbst eine scharfe Rezession erleben. Aber sie verfügen über die finanziellen Mittel, um die Krise zu überwinden, indem sie ihren Unternehmen und Arbeitslosen großzügige Hilfe leisten. Sie wären in der Lage, auch den bedürftigsten Mitgliedstaaten eine gewisse Unterstützung zu gewähren.

## Europäische Union oder Euroraum?

Eine erste Frage ist, ob die Corona-Krise Solidarität innerhalb der EU oder des Euroraums erfordert. Während der Finanzkrise könnte man argumentieren, dass die Mitgliedschaft im Euroraum selbst eine Quelle der Schwierigkeiten für Italien und Spanien war, weil sie sich angesichts des Drucks auf ihren Finanzmärkten nicht an ihre nationalen Zentralbanken wenden konnten, um Liquidität zu erhalten. Der Ursprung dieser Krise ist vollkommen anders. Von einer unvorhergesehenen Epidemie getroffen zu werden, hat nichts mit der Mitgliedschaft in der Europäischen Währungsunion (EWU) zu tun. Es gibt also ein starkes Argument, die gegenwärtige Situation als einen Fall zu betrachten, der Solidarität auf EU-Ebene erfordert.

Die 19 Euro-Länder stellen 85 % der Wirtschaft der 27 EU-Mitglieder. Ob die Maßnahmen auf der Ebene der EU oder auf der Ebene der EWU ansetzen, ist aber trotzdem wichtig, weil der institutionelle Rahmen sich unterscheidet. Der Kernpunkt ist, dass das Euro-Währungsgebiet nicht über Mechanismen für Transfers verfügt, sondern nur Kredite bereitstellen kann. Dies ist durchaus entscheidend, denn der gesamte institutionelle und juristische Rahmen des Euroraums sollte Transfers zwischen Ländern ausschließen. Der Vertrag von Maastricht bestimmt ausdrücklich, dass weder die EU noch einzelne Mitgliedstaaten für die Schulden eines andern Mitgliedstaats verantwortlich sind. Die EWU wurde in Deutschland unter der Voraussetzung angepriesen, dass eine gemeinsame Geldpolitik nicht bedeutet, dass die deutschen Steuerzahler für die Schulden anderer Länder haften würden. Über die Institutionen des Euroraums ließen sich also keine Transfers organisieren.

Im Gegensatz dazu verfügt die Europäische Union über einen Haushalt, mit dem Mittel von den reicheren in die ärmeren und weniger entwickelten Teile der EU transferiert werden sollen. Offiziell ist dies Gegenstand der Regionalpolitik, d. h. diese Transfers sollen bedürftigen Regionen helfen, unabhängig davon, in welchem Staat sie liegen. In Wirklichkeit richtet sich die Mittelzuweisung jedoch auf die nationale Ebene. Arme Regionen in reichen Ländern erhalten nur einen Bruchteil der Mittel im Vergleich zu ebenso armen Regionen in weniger wohlhabenden Ländern. Der EU-Haushalt sieht auch ausdrücklich große Transfers an Landwirte unter dem Deckmantel der Gemeinsamen Agrarpolitik vor, die immer noch etwa ein Drittel des EU-Haushalts ausmachen (Gros et al., 2020).

Es hatte auch einen Versuch gegeben, das Euro-Währungsgebiet mit einem Element auszustatten, das es erlauben würde, asymmetrische Schocks aufzufangen. Dies war die Idee hinter dem sogenannten „Haushaltsinstrument für Konvergenz und Wettbewerbsfähigkeit“ (Budgetary instrument for convergence and competitiveness, BICC). Die für dieses potenzielle Instrument vorgesehenen Beträge waren bisher so gering (weniger als 3 Mrd. Euro pro Jahr), dass es keine wesentlichen Auswirkungen haben kann. Es ist interessant, dass man jetzt nicht die Gelegenheit genutzt hat, dieses Instrument einfach erheblich auszuweiten.

### Zuschüsse oder Darlehen?

Die Frage des institutionellen Rahmens kreuzt sich mit der Frage, auf welche Art Hilfe gewährt werden sollte: Darlehen oder Zuschüsse (Transfers)? Dies ist die zweite Schlüsselfrage. Am Anfang der Krise wurde argumentiert, dass die zusätzlichen Ausgaben aufgrund des Coronavirus-Schocks durch gemeinsame Anleihen finanziert werden sollten. Anfangs forderten deshalb eine Reihe von Staaten, „Coronabonds“ zu schaffen. Dies wären Schuldtitel gewesen, die einzelne Mitgliedstaaten ausgeben, aber von allen garantiert wären. Diese Anleihen hätten niedrige Zinsen, weil sie durch die Garantie besonders der starken Mitgliedstaaten gestützt würden.

Eine andere Idee war es, den besonders betroffenen Ländern zu ermöglichen, bei dem Europäischen Stabilitätsmechanismus (ESM) günstige Kredite aufzunehmen. Aber das würde das Hauptproblem für die haushaltspolitisch gefährdeten und strukturschwachen Länder nicht lösen, denn sie haben bereits zu hohe Schulden. Die italienische Regierung würde einen niedrigeren Zinssatz für diesen Teil ihrer Schulden zahlen, aber die Gesamtverschuldung Italiens würde weiter steigen. Darüber hinaus würde der relativ geringe Gewinn in Form von Zinsersparnissen durch Coronabonds oder ESM-Darlehen wahrscheinlich durch höhere Kosten der verbleibenden Schulden (noch 135 % des Bruttoinlandsprodukts) ausgeglichen, da diese Altschulden effektiv den Krediten der EU untergeordnet würden. Eine Erhöhung der gesamten Staatsverschuldung Italiens wäre gefährlich, da ein höherer Schuldenstand in der Regel eine höhere Risikoprämie erfordert, was zu einer Spirale führen kann, unter der höhere Schulden zu einer höheren Risikoprämie führen, was zu höheren Defiziten und sogar höheren Schulden führt.

Aus diesem Grund brauchen die Länder im Süden, die derzeit am stärksten vom Coronavirus betroffen sind, Zuschüsse und keine Kredite (Gros, 2020). Diese Einsicht hat sich durchgesetzt und führte letztendlich zu dem gemeinsamen Vorschlag von Macron und Merkel.

### Deutsch-französisches Abkommen über einen „Sanierungsfonds“

Mitte Mai 2020 haben Bundeskanzlerin Merkel und Präsident Macron eine gemeinsame deutsch-französische Initiative für einen 500-Mrd.-Euro-Wiederaufbausfonds zur Emission von Anleihen und zur Finanzierung von Ausgaben in den am stärksten betroffenen Ländern und Sektoren vorgelegt. Angesichts der rechtlichen Beschränkungen des EU-Haushalts kann dies erreicht werden, indem die sogenannte Ausgabenobergrenze für den EU-Haushalt für die nächsten drei Jahre von etwa 1 % auf 2 % des Bruttonationaleinkommens angehoben wird. Dieser Vorschlag für einen Sanierungsfonds würde zusätzliche Ausgaben in Höhe von rund 165 Mrd. Euro pro Jahr ermöglichen und sich über drei Jahre auf 500 Mrd. Euro summieren. Der Fonds würde somit in den EU-Haushalt integriert und in erster Linie die Ausgaben im Gesundheitssektor finanzieren, aber möglicherweise auch den grünen Übergang unterstützen und den von der aktuellen Krise am stärksten betroffenen Ländern helfen.

Diese deutsch-französische Initiative ist ein wichtiger politischer Schritt. Frankreich und Deutschland können den Fonds nur anderen Mitgliedstaaten vorschlagen, aber da sie effektiv die beiden Lager repräsentieren, die bisher in einer Pattsituation waren, ist es unwahrscheinlich, dass ein anderer Mitgliedstaat es vollständig blockieren wird. Was den Vorschlag politisch attraktiv macht, ist, dass niemand heute zahlen muss. Stattdessen werden zusätzliche Ausgaben mit Anleihen finanziert, die während der Laufzeit des nächsten Finanzrahmens der EU, d.h. nicht vor 2028, nicht zurückgezahlt werden müssen. Der eigentliche Streit darüber, wer die zusätzlichen „Corona"-Ausgaben bezahlt, wird somit verschoben. Für die nächsten sieben Jahre wird kein Land gezwungen sein, seinen Beitrag zum EU-Haushalt zu erhöhen. Der Widerstand von Ländern wie Österreich, den Niederlanden, Dänemark und Schweden wird deshalb mehr von Prinzipien als von Eigeninteresse getragen.

Die EU-Kommission hat den deutsch-französischen Vorschlag aufgegriffen und etwas erweitert, indem sie weitere 250 Mrd. Euro für langfristige Kredite bereitstellen möchte. Sie schlägt also einen „nächste Generation"-Fonds von 750 Mrd. Euro vor, um damit Mitgliedstaaten zu unterstützen nicht nur um die jetzige Krise zu bewältigen, aber auch um ihnen bei der Digitalisierung und der Energiewende zu helfen.

## Wie geht es weiter?

Zunächst einmal müssen alle Mitgliedstaaten dem Plan der EU-Kommission zustimmen. Dies wird nicht einfach sein, denn es gibt noch viele praktische und politische Hürden zu überwinden. In normalen Zeiten würde es Jahre brauchen, um eine Einigung aller 27 Mitgliedstaaten zu erzielen. Zum Glück ist die Lage aber so angespannt, dass es diesmal wohl nur Monate dauern wird. Der offizielle Zeitplan sieht vor, dass schon im Juli 2020 über die wesentlichen Parameter eine Einigung erreicht werden sollte, wie über den Gesamtumfang des Pakets, welcher Anteil Zuschüsse und nach welchen Kriterien die Finanzmittel verteilt werden sollten. Idealerweise könnten also die ersten Finanzmittel schon in diesem Jahr fließen.

Das eigentliche Problem ist dabei der Zielkonflikt zwischen Schnelligkeit und Genauigkeit. Einerseits sollten die Mittel so schnell wie möglich eingesetzt werden, um auch schnell zu wirken und das Ausmaß der Krise zu lindern. Andererseits möchte man die Finanzmittel auch nicht nach dem Gießkannenprinzip verteilen, sondern sinnvoll einsetzen. Um eine vernünftige Verwendung der Mittel zu garantieren bedarf es aber Zeit. Auch hier wird es einen Kompromiss geben müssen, mit einigen Mitteln, die sofort an besonders hart getroffene Länder fließen und anderen, die erst in den nächsten Jahre ausgezahlt werden, wenn ein überzeugendes Programm vorliegt.

Diese Corona-Paket steht nicht allein, sondern es muss auch in das EU-Budget eingebettet werden. Das Budget der EU wird über den mehrjährigen EU-Finanzrahmen bestimmt, der zufällig dieses Jahr wieder neu für 2021 bis 2027 bestimmt werden muss. Aber das „nicht-Corona"-Budget der EU sollte diesmal keine große Probleme bereiten. Bestehende Ausgaben für Landwirtschaft und Regionalfonds (ein Großteil davon mit zweifelhafter europäischer Wertschöpfung) werden aller Wahrscheinlichkeit nach mehr oder weniger wie bisher fortgesetzt, ebenso wie die Beiträge der Mitgliedstaaten zur EU. Die zusätzlichen Ausgaben im Zusammenhang mit der Corona-Krise werden ja durch von der EU ausgegebene Schuldtitel finanziert.

Diese zukünftigen EU-Anleihen sind nicht ganz die gleichen wie die berühmten Corona-Anleihen, gegen die Deutschland bis vor kurzem so stark Widerstand geleistet hat. In diesem Fall wird die EU als eigenständiger Schuldner auftreten – nicht die Mitgliedstaaten. Dies ist nicht ganz neu. Es sind bereits einige kleine Beträge von EU-Anleihen ausstehend, die mit einem Kreditrating von AAA bewertet wurden – dies aber hauptsächlich, weil die Beträge so gering sind. Es bleibt abzuwarten, wie die Finanzmärkte (und Ratingagenturen) auf die EU reagieren werden, die jetzt 500 Mrd. Euro emittiert. Die Einzelheiten darüber, welche Garantien gegeben werden müssen, um sicherzustellen, dass Anleger diese Anleihen kaufen, werden Anwälte und Ratingagenturen für einige Zeit beschäftigen. Am Ende wird es um die Frage gehen, ob die Märkte die EU als eine tragfähige politische Institution betrachten, die ihre eigene Finanzierung sicherstellen kann, ohne sich auf die ultimative Garantie ihrer stärksten Mitglieder verlassen zu müssen (Kramer, 2020).

Die politischen und finanziellen Hürden können überwunden werden. Aber das allein wird nicht genügen. Langfristig wird entscheidend sein, wie das Geld ausgegeben wird und ob sich gerade die Länder wieder erholen, die jetzt besonders von der Krise betroffen sind. Wenn dies der Fall ist, wenn man sieht, dass die EU einen wichtigen Beitrag zur Bewältigung dieser Jahrhundertkrise geleistet hat, wird Europa gestärkt aus dieser Prüfung hervorgehen.

Dr. Daniel Gros ist Direktor des Centre for European Policy Studies (CEPS) in Brüssel, Belgien.


Literatur

Gros, D., G. Barci und J. Núñez Ferrer (2020), Towards a new MFF — New priorities and their impact on Italy, CEPS Research Paper, 2020, https://www.ceps.eu/ceps-publications/towards-a-new-mff/ (8. Juni 2020).
Gros, D. (2020), EU solidarity in exceptional times: Corona transfers instead of Coronabonds, Voxeu.org, 5. April 2020, https://voxeu.org/article/corona-transfers-instead-coronabonds (8. Juni 2020).
Kramer, M. (2020), Next Generation EU bonds might face a credit-rating challenge, CEPS, Commentary, Published on 04 June 2020, https://www.ceps.eu/next-generation-eu-bonds-might-face-a-credit-rating-challenge/ (8. Juni 2020).
